# Inosine‐Triphosphate‐Pyrophosphatase Activity as a Potential Predictor of Methotrexate Remission in Juvenile Idiopathic Arthritis

**DOI:** 10.1002/art.70097

**Published:** 2026-03-22

**Authors:** Sofia Sindici Forgiarini, Marianna Lucafò, Debora Curci, Davide Selvestrel, Valentina Moressa, Sofia Pagarin, Barbara Bellich, Martina Franzin, Alberto Tommasini, Mara L. Becker, Laura B. Ramsey, Susan D. Thompson, Carl D. Langefeld, Federica Corba, Edoardo Marrani, Gabriele Simonini, Gabriele Stocco, Giuliana Decorti, Andrea Taddio, Serena Pastore

**Affiliations:** ^1^ Department of Medicine, Surgery and Health Sciences University of Trieste Trieste Italy; ^2^ Department of Life Sciences University of Trieste Trieste Italy; ^3^ Institute for Maternal and Child Health IRCCS Burlo Garofolo Trieste Italy; ^4^ International Centre for Genetic Engineering and Biotechnology Trieste Italy; ^5^ Department of Pediatrics Duke University School of Medicine Durham North Carolina; ^6^ Department of Pediatrics Children's Mercy Kansas City Kansas City Missouri; ^7^ Department of Pediatrics University of Missouri‐Kansas City; ^8^ Department of Pediatrics, University of Cincinnati College of Medicine, Division of Human Genetics Cincinnati Children's Hospital Medical Center Cincinnati Ohio; ^9^ Department of Biostatistics and Data Science Wake Forest School of Medicine Winston‐Salem North Carolina; ^10^ Pediatric Rheumatology Unit Meyer Children's Hospital IRCCS Florence Italy

## Abstract

**Objective:**

Methotrexate (MTX) is the first‐line therapy for juvenile idiopathic arthritis (JIA), but up to 40% of patients do not respond to it. Low inosine triphosphate pyrophosphatase (ITPA) activity has been associated with reduced clinical remission. We investigated the role and underlying mechanisms of ITPA in vitro.

**Methods:**

ITPA enzymatic activity was measured by high‐performance liquid chromatography with ultraviolet detection in erythrocyte lysates from patients with JIA (enrolled in Trieste, Italy; Florence, Italy; and Kansas City, Missouri) receiving weekly MTX (10–15 mg/m^2^) for at least 12 months. Remission was evaluated by Wallace et al criteria. *ITPA* rs1127354, which reduces enzyme activity, was genotyped. Immortalized human hepatocytes were transfected with either an *ITPA*‐containing or empty plasmid to evaluate cytotoxicity (MTT), ADORA_2A_ expression (quantitative polymerase chain reaction), intracellular cyclic AMP (cAMP) (enzyme‐linked immunosorbent assay), PKA‐Cα expression (immunoblot), and extracellular adenosine metabolites (liquid chromatography–tandem mass spectrometry).

**Results:**

A total of 195 patients with JIA (mean age: 8 years, range: 0–22 years, 74% female participants) were enrolled. Patients reaching remission (48%) had higher ITPA activity than nonresponders during therapy and at treatment onset (meta‐analysis: *P* = 0.0004 and *P* = 0.007, respectively). Predictive cutoffs for overall and onset measurements were identified: 181.9 and 177.8 nmol of inosine monophosphate per hour. None of the patients with variant *ITPA* rs1127354 achieved remission (Fisher's exact test *P* = 0.0063); however, 48% of wild‐type patients failed to respond. ITPA‐overexpressing cells showed elevated MTX sensitivity, cAMP levels, PKA‐Cα expression, and hypoxanthine (all *P* < 0.05, two‐way analysis of variance).

**Conclusion:**

ITPA activity is a promising predictive biomarker for MTX response in JIA, outperforming *ITPA* rs1127354 genotyping. Its predictive value is evident at treatment initiation, supporting its use for personalized treatment. Mechanistically, higher enzyme activity may enhance MTX efficacy through the adenosine/inosine‐ADORA_2A_ pathway.

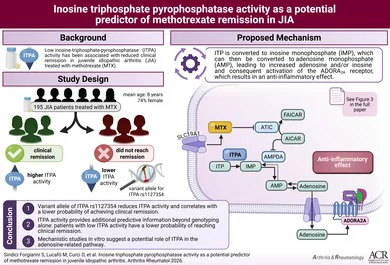

## INTRODUCTION

Juvenile idiopathic arthritis (JIA) is the most common chronic rheumatic disease in childhood and is characterized predominantly by immune‐mediated inflammatory arthritis with a heterogenous clinical presentation.^1^, ^2^ Low‐dose methotrexate (MTX), which is highly effective both as monotherapy and in combination with biologic drugs, is a first‐line therapy in JIA.^3^ As a folate analog, MTX modulates pathways that involve folates, including the de novo synthesis of purines and pyrimidines by inhibition of the activities of dihydrofolate reductase (DHFR) and thymidylate synthetase.^4^ However, folic acid administration does not interrupt the anti‐inflammatory effect of MTX, suggesting additional mechanisms behind its action in JIA.^3^

The most widely accepted mechanism for MTX's therapeutic response in JIA is the accumulation of adenosine, mainly caused by 5‐aminoimidazole‐4‐carboxamide ribonucleotide transformylase inhibition.^3^ Extracellular adenosine subsequently binds its receptors, the ADORAs, and the consequent downstream signaling of ADORA_2A_ stimulation inhibits nuclear factor κB (NF‐κB), a key regulator of inflammatory responses.^3^, ^5^, ^6^, ^7^ Indeed, the administration of two adenosine receptor antagonists, caffeine and theophylline, nullifies the MTX effect in the rat adjuvant arthritis model of rheumatoid arthritis (RA).^8^ Moreover, patients with RA who consume large quantities of caffeine have been shown to have reduced responsiveness to MTX.^9^

Despite MTX safety, effectiveness, and low cost, up to 40% of patients do not respond to therapy, which leads to disease progression and permanent joint damage.^10^ Therefore, identifying biomarkers of MTX response is crucial to direct patients toward the optimal therapeutic option.^3^ Inosine triphosphate pyrophosphatase (ITPA) activity measured during therapy is correlated with MTX response, evaluated as remission^11^: patients with low ITPA activity presented lower remission probability.^11^ ITPA is an essential enzyme that preserves nucleotide homeostasis by hydrolyzing noncanonical triphosphate purine nucleotides, such as (deoxy)inosine triphosphate (ITP) and (deoxy)xantosine triphosphate, into their monophosphate derivatives.^12^ Inosine monophosphate (IMP) can be converted to AMP, which can then be further converted to adenosine.^11^ Therefore, the aim of this study was to evaluate the correlation of ITPA activity, measured both at the onset of MTX therapy and during treatment, with the probability of remission, expanding the previous cohort of patients.^11^ Additionally, we aimed to investigate in vitro molecular mechanisms linking ITPA activity with MTX response.

## MATERIALS AND METHODS

### Patients and study design

Children meeting International League of Associations for Rheumatology criteria for JIA, receiving or about to start MTX for active arthritis, were enrolled from three major pediatric rheumatology hospitals (IRCCS Burlo Garofolo Children's Hospital and Meyer Children's Hospital IRCCS in Italy and Children's Mercy Hospital of Kansas City in Missouri). Eligible patients were either treatment‐naïve or had not received MTX for at least 10 months before enrollment. Patients with systemic JIA or receiving concomitant biologic therapy were excluded. The study was conducted according to the Declaration of Helsinki and approved by the Regional Ethics Committee of Friuli Venezia Giulia (CEUR‐2018‐Em‐137‐BURLO, September 5th, 2018). Fully informed parental consent and child assent, when appropriate, were obtained. Demographic and clinical data, as well as venous blood samples, were collected at baseline (up to 4 weeks before treatment), at 6 months, and then every 3 months, with a minimum follow‐up of 12 months. The timing of sample collection was standardized across all study participants; however, not all time points were available for every patient. Analyses using the mean of all time points were performed on available samples. For the analyses at therapy onset, patients without samples and clinical data collected before MTX initiation were excluded to minimize potential bias. Therapy onset was defined as the visit when MTX was prescribed. Weekly MTX (10–15 mg/m^2^) was administered orally or subcutaneously. Clinical remission was defined according to Wallace et al criteria^13^ as a continuous six‐month period of clinically inactive disease (CID) while on MTX therapy, beginning within one year of starting therapy. CID was defined as absence of joints with active disease, systemic symptoms, and uveitis; erythrocyte sedimentation rate and/or C‐reactive protein within normal range (if both were tested); physician's global assessment at the best score; and morning stiffness less than 15 minutes. During this period, patients received no other drugs (biologics, nonsteroidal anti‐inflammatory drugs, oral or intra‐articular steroids). Race information was obtained from medical records or self‐reported using standard categories.

### Measurement of ITPA activity

ITPA activity was measured in erythrocyte lysates by quantifying IMP, the ITP hydrolysis product, using high‐performance liquid chromatography (HPLC) with ultraviolet (UV) detection (Agilent 1200 HPLC with UV detector, Agilent Technologies, Milan, Italy), as previously reported.^14^ The detailed protocol is in the Supplementary Material.

### Genotyping of *ITPA* rs1127354

Total genomic DNA was extracted from peripheral blood by a commercial kit (Sigma‐Aldrich, Milan, Italy) and stored at −20°C. Genotyping used a TaqMan assay (identification: C_16218146_10, Applied Biosystems, Foster City, California).^11^, ^14^

### Immortalized cell lines and cell culture

The immortalized human hepatic IHH cell line was stably transfected with pCMV6 plasmid vectors, empty (IHH MOCK) or containing the *ITPA* complementary DNA (cDNA) sequence (IHH ITPA).^15^ IHH cell lines were maintained as described in the Supplementary Material.

### MTT cytotoxicity test

To evaluate the cytotoxicity of MTX on cell lines, the 3‐(4,5‐dimethylthiazol‐2‐yl)‐2,5‐diphenyltetrazolium bromide (MTT) assay was performed. IHH MOCK and ITPA cells were seeded at 5 × 10^3^ cells/well in 96‐well plates and exposed to six MTX concentrations (3.125 nM, 25 nM, 200 nM, 600 nM, and 200 µM) in DMEM‐HG for 72 hours. Details are provided in the Supplementary Material.

### Total RNA isolation and reverse transcription

Total RNA was extracted from IHH MOCK and IHH ITPA cell lines using TRIzol reagent (Thermo Scientific, Carlsbad, California) and the PureLink RNA Mini kit (Thermo Fisher Scientific, Milan, Italy). Concentration and purity were determined by NanoDrop 2000 (EuroClone, Milan, Italy). Reverse transcription was conducted with the High‐Capacity RNA‐to‐cDNA Kit (Applied Biosystem, Foster City, California).

### Quantitative real‐time polymerase chain reaction

ADORA_2A_ expression was evaluated by SYBR green‐based real‐time polymerase chain reaction (RT‐PCR) using the CFX96TM RealTime Detection System‐C1000 Thermal Cycler (Bio‐Rad Laboratories, Hercules, California). Real‐time PCR, performed in triplicate, used KiCqStart SYBR Green qPCR ReadyMix (Sigma‐Aldrich, Milan, Italy) and gene‐specific forward and reverse primers (Sigma‐Aldrich, Milan, Italy). Thermal cycling conditions were set as described in the Supplementary Material.

### Intracellular levels of cyclic AMP

Intracellular cyclic AMP (cAMP) levels were assessed with the cyclic AMP enzyme‐linked immunosorbent assay kit (Cayman Chemical, Ann Arbor, Michigan). IHH MOCK and IHH ITPA cells, seeded in six‐well plates at densities of 7.5 × 10^5^ cells/well for the 24‐hour treatment and 4.8 × 10^5^ cells/well for the 48‐hour one, were exposed to MTX (25 nM, 200 nM). cAMP concentrations, normalized to total protein content, were expressed as a percentage relative to untreated cells.

### Western blot analysis

Cells, seeded in six‐well plates at densities of 7.5 × 10^5^, 4.8 × 10^5^, and 3.3 × 10^5^ cells/well for 24‐, 48‐, and 72‐hour treatments, respectively, were exposed to MTX (25 nM, 200 nM). Protocol details, including lysis procedures, are provided in the Supplementary Material.

### Quantification of adenosine, inosine, hypoxanthine and uric acid in cell supernatants through LC‐MS/MS

Cells, seeded in 24‐well plates at a density of 1.6 × 10^5^ cells/well, were treated for 48 hours with 25 and 200 nM of MTX. To preserve adenosine levels at the end of the treatment, a stop solution (see the Supplementary Material) was added 30 minutes before the end of incubation. Cell supernatants were collected, filtered using Amicon Ultra centrifugal filters, MWCO 10 kDa (Millipore, Milan, Italy), and stored at −80°C. Supernatants were thawed immediately before LC‐MS/MS analysis and analyzed by Ultra‐High‐Pressure Liquid Chromatography Vanquish Neo UHPLC instrument (Thermo Fisher Scientific, Milan, Italy) coupled with mass spectrometry Exploris 240 (Thermo Fisher Scientific, Milan, Italy). Chromatographic separation and mass spectrometry acquisition followed the Supplementary Material protocol.

### Statistical analysis

Analyses were performed using GraphPad Prism and R (v3.6.1–4.5.0). ITPA activity differences across clinical sites were assessed by the Kruskal‐Wallis test with Dunn's post‐hoc test (Bonferroni corrected). Sex associations were evaluated by logistic regression (clinical remission) and the Wilcoxon rank‐sum test (ITPA activity). Remission–ITPA activity association was tested by logistic regression, adjusting for clinical site via multivariate models and meta‐analysis (weighted Z‐method). Longitudinal ITPA activity changes were assessed by linear mixed‐effects models with random intercepts (patient, site). The role of clinical site on remission was tested via likelihood ratio test. Receiver operating characteristic (ROC) curve analysis (minimum distance to [0,1]) identified ITPA thresholds predictive of remission. ITPA activity was compared across rs1127354 genotypes and between wild‐type (WT) patients (remission vs nonremission) using the Wilcoxon test. Genotype–remission association was tested by Fisher's exact test with Haldane–Anscombe correction. In vitro data were analyzed by two‐way analysis of variance (ANOVA) with Sidak's test. ITPA activity is expressed as mean ± SD. Data from all other experiments are shown as mean ± SEM; *P* < 0.05 was considered significant. In vitro assays were based on at least three biologic replicates. The data analyzed for the current study are available from the corresponding author on reasonable request.

## RESULTS

### Patients enrolled and clinical remission

Demographic and clinical data of 195 patients with JIA, treated with MTX between 2000 and 2022, are reported in Table 1. A total of 134 patients were enrolled in Italy (112 in Trieste at IRCCS Burlo Garofolo and 22 in Florence at IRCCS Meyer) and 61 in the United States (in Kansas City, Missouri, at Children's Mercy). Clinical remission was achieved by 48% of patients (93 of 195). Although 74% of patients were female, sex was not significantly associated with either clinical remission (logistic regression: *P* = 0.55) or ITPA activity (Wilcoxon rank‐sum test: *P* = 0.60). Racial information was collected for 176 of 195 patients; for the remaining 19 patients this information was not available.

**Table 1:** Patients’ demographic and clinical data*

Characteristic	Overall	Remission	No remission
Number of cases, N/n (%)	195	93 (48)	102 (52)
Female, n (%)	145 (74)	71	74
Male, n (%)	50 (26)	22	28
White, n (%)	176 (90)		
Unknown, n (%)	19 (10)		
JIA subtypes at the beginning of therapy with MTX, n (%)			
Oligoarticular persistent	97 (50)	53	44
Oligoarticular extended	8 (4)	1	7
Polyarticular RF –	65 (33)	28	37
Polyarticular RF +	8 (4)	2	6
Enthesitis related arthritis	9 (5)	6	3
Psoriatic	7 (4)	2	5
Undifferentiated	1 (0.5)	1	0
Age at the start of MTX, mean and range, y	8, 0–22	6, 0–22	10, 0–21

* JIA, juvenile idiopathic arthritis; MTX, methotrexate; RF, rheumatoid factor.

### Clinical remission and ITPA activity

The association of ITPA activity with remission was evaluated considering all time points during therapy and only the measurement at therapy onset. The all‐time points analysis included all 195 patients, whereas the baseline‐only analysis involved 97 patients with JIA (43 from Trieste, 18 from Florence, and 36 from Kansas City) because samples at therapy onset were not available for the remaining patients.

A significant association was observed between ITPA activity and clinical remission when considering all time points (logistic regression: *P* = 0.0012, odds ratio [OR] = 1.01, 95% confidence interval [CI] 1.002–1.009), as well as when ITPA activity was measured at therapy onset alone (logistic regression: *P* = 0.01, OR = 1.01, 95% CI 1.002–1.011). A likelihood ratio test did not show a significant effect of the clinical site on remission (all time points: *P* = 0.57, therapy onset: *P* = 0.25).

A significant difference in ITPA activity emerged among clinical sites, both when considering all measurements during therapy and only those at therapy onset (all measurements: Kruskal‐Wallis test *P* = 4.62 × 10^−5^, therapy onset: Kruskal‐Wallis test *P* = 0.025). In particular, patients at the IRCCS Burlo Garofolo in Trieste showed lower activity than those from the other hospitals when all time points are considered (Dunn's post test with Bonferroni correction: Trieste vs Florence *P* = 2.0 × 10^−4^, Trieste vs Kansas City *P* = 3.0 × 10^−3^) (See Supplementary Figure 1). A significant difference was also observed when considering only the measurements at therapy onset (Dunn's post test with Bonferroni correction: Trieste vs Florence *P* = 5.4 × 10^−3^, Trieste vs Kansas City *P* = 3.1 × 10^−3^) (see Supplementary Figure 2).

**Figure 1:**
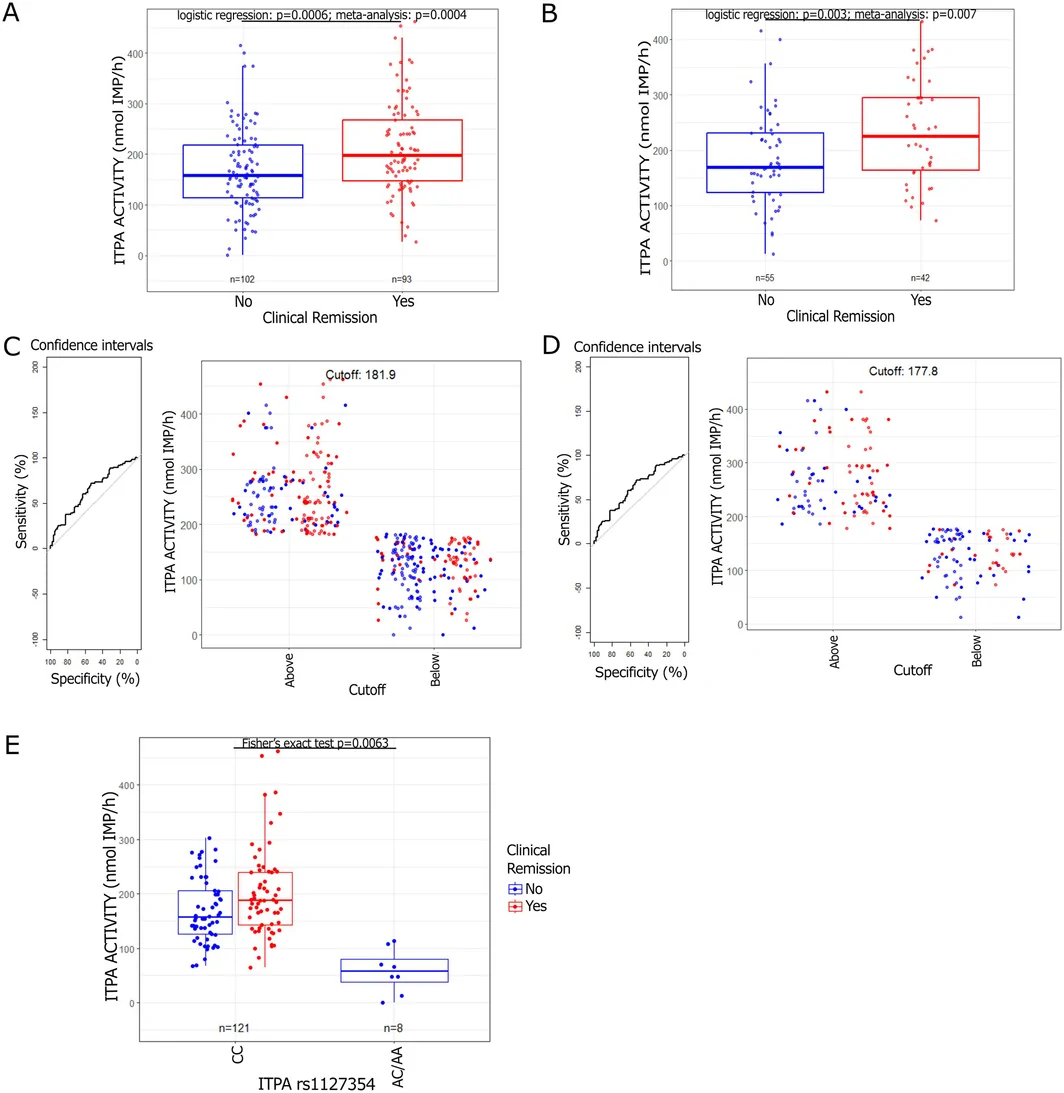
ITPA activity in patients with JIA, considering their clinical remission. (A) Boxplots showing mean ITPA activity at all time points during therapy, comparing patients who did not achieve remission with those who did. (B) Boxplots showing mean ITPA activity at therapy onset, comparing patients who did not achieve remission with those who did. (C) ROC curve and boxplot for ITPA activity measured during therapy. Patients with JIA are stratified by the predictive cutoff of 181.9 nmol IMP/h, displaying their remission status. (D) ROC curve and boxplot for ITPA activity measured at therapy onset. Patients with JIA are stratified by the predictive cutoff of 177.8 nmol IMP/h, displaying their remission status. (E) Boxplot showing ITPA activity during treatment and clinical remission status for rs1127354 genotype (WT vs patients carrying the variant) in patients with JIA. Panels were created using Inkscape. IMP, inosine monophosphate; ITPA, inosine triphosphate pyrophosphatase; JIA, juvenile idiopathic arthritis; ROC, receiver operating characteristic; WT, wild‐type.

Despite these differences, the association between ITPA activity and remission remained significant for overall measurements during treatment after adjustment for clinical site (logistic regression: *P* = 0.0006; meta‐analysis: *P* = 0.0004) and was also confirmed for those at therapy onset (logistic regression: *P* = 0.003; meta‐analysis: *P* = 0.007). In detail, patients with JIA who reached remission had higher ITPA activity than those who did not, both for all time points (212.1 ± 90.8 vs 169.6 ± 81.5 nmol IMP/h) (Figure 1A) and for the therapy onset (232.4 ± 92.0 vs 183.7 ± 84.3 nmol IMP/h) (Figure 1B). Supplementary Tables 1 and 2 summarize ITPA activity stratified by clinical site, including number of patients per group, mean ITPA activity for remission and nonremission groups, logistic regression *P* values, OR, and 95% CI.

ITPA activity was not significantly different between therapy onset and subsequent treatment time points (203.3 ± 91.7 vs 193.30 ± 98.5 nmol IMP/h, linear mixed‐effects model with random intercepts for patient and clinical site: *P* = 0.38) (Supplementary Figure 3). Considering only those patients who achieved remission, there was no significant difference between ITPA activity at onset and during therapy (229.8 ± 94.8 vs 211.9 ± 106.4 nmol IMP/h, same model: *P* = 0.65) (Supplementary Figure 4).

### Potential cutoffs of ITPA activity for remission

ROC curve analysis was used to define a cutoff value of ITPA activity to discriminate between patients achieving remission and those who did not. For all time points, the calculated cutoff was 181.9 nmol IMP/h, yielding a sensitivity of 60% and a specificity of 63% (Figure 1C). Specifically, 60% (56 of 94) of patients above the determined cutoff achieved clinical remission, compared with 37% (37 of 101) of those below the threshold.

For the therapy onset, the calculated cutoff was 177.8 nmol IMP/h, corresponding to a sensitivity of 67% and a specificity of 58% (Figure 1D). Specifically, 55% (28 of 51) of patients above the determined cutoff achieved clinical remission, compared with 30% (14 of 46) of those below the threshold.

### Clinical remission and genotyping

*ITPA* rs1127354 was genotyped in 129 patients with JIA. Five patients from the Italian cohort and the American cohort were excluded because of lack of available DNA. In the analyzed cohort, the polymorphism was in the Hardy–Weinberg equilibrium, and its frequency agrees with its distribution in the White population, with minor allele frequency of 3.9%. In detail, 8 of 129 patients with JIA carried the variant. As shown in Figure 1E, ITPA activity was higher in WT patients compared with those carrying the variant (Wilcoxon rank‐sum test: *P* = 6.2 × 10^6^, 186.0 ± 73.6 vs 58.2 ± 40.1 nmol IMP/h).

None of the patients with the variant reached clinical remission (Fisher's exact test *P* = 0.0063, corrected OR = 18.45, 95% CI 1.04–326.82). In contrast, 63 WT patients (52%) achieved remission. Among WT patients, ITPA activity was still associated with a greater likelihood of achieving remission (Wilcoxon rank‐sum test, *P* = 0.044).

### Role of ITPA overexpression in modulating MTX sensitivity in IHH cells

**Figure 2:**
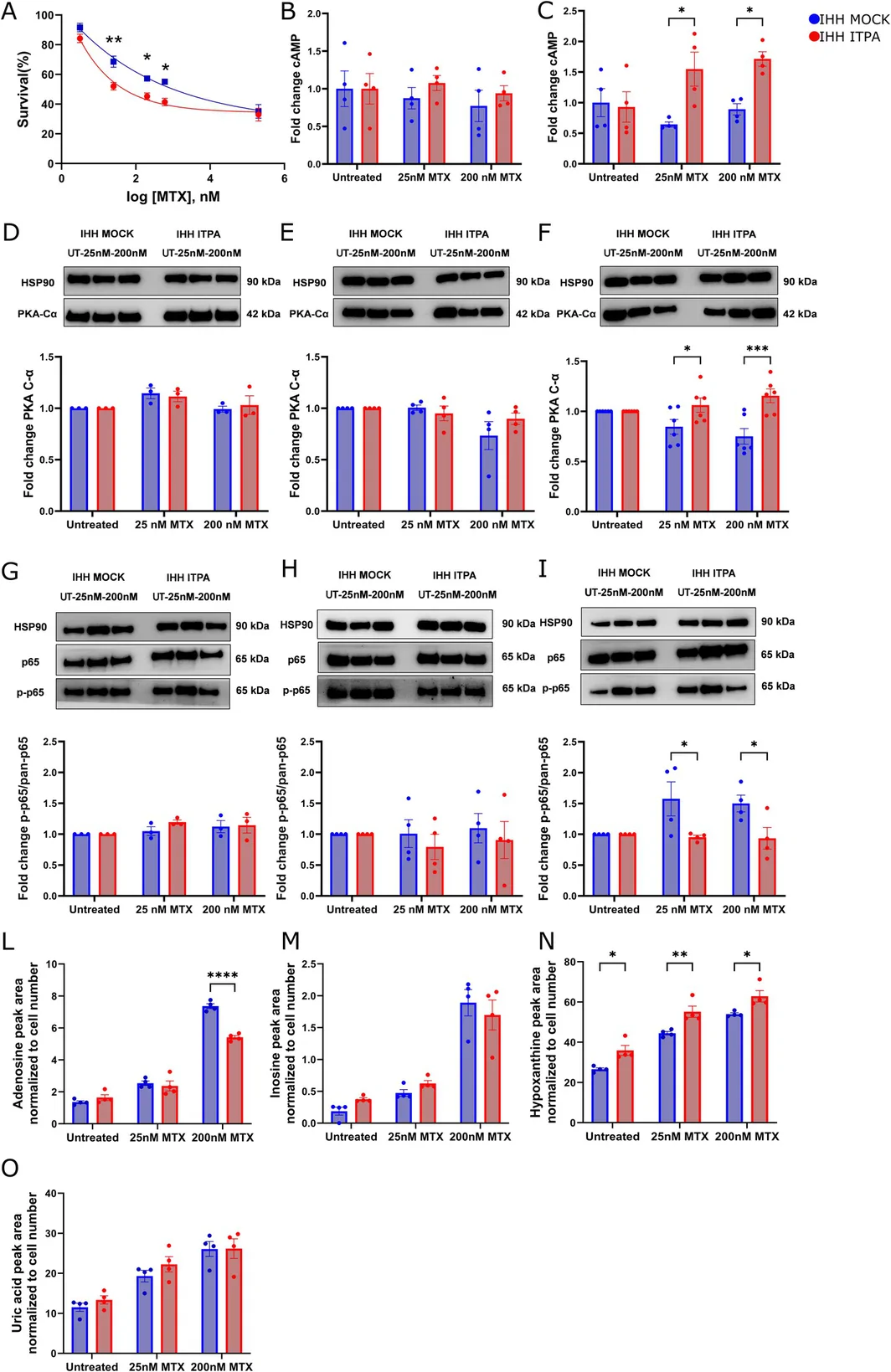
MTX cytotoxic and molecular effects treatment on IHH MOCK and ITPA cell lines. Western blot analyses were normalized to HSP90; fold change was calculated relative to the corresponding untreated condition. Data (mean ± SEM) were analyzed by two‐way ANOVA with Sidak's post‐test (* = *P* < 0.05, ** = *P* < 0.01, *** = *P* < 0.0001, **** = *P* < 0.0001). (A) Cytotoxic effects of 72‐hour MTX treatment (n = 3) (IHH ITPA vs IHH MOCK at 3.125, 25, 200, 600, 200 µM). (B) and (C) cAMP levels after 24 (n = 4) and 48 (n = 4) hours of MTX treatment (25 nM, 200 nM) (IHH ITPA vs IHH MOCK at 0, 25, 200 nM). (D–F) PKA‐Cα expression after 24 (n = 3), 48 (n = 4) and 72 (n = 6) hours of treatment in cell lysates from untreated (NT) and MTX‐treated (25 nM, 200 nM) cells (IHH ITPA vs IHH MOCK at 0, 25, 200 nM). (G–I) Phosphorylated form of p65‐NF‐κB after 24 (n = 3), 48 (n = 4), and 72 (n = 3) hours of MTX treatment in cell lysates from untreated (NT) and MTX‐treated (25 nM, 200 nM) cells (IHH ITPA vs IHH MOCK at 0, 25, 200 nM) (L–O) LC‐MS/MS quantification of adenosine, inosine, hypoxanthine, and uric acid in the supernatant after 48‐hour MTX (n = 3) (IHH ITPA vs IHH MOCK at 0, 25, 200 nM). Panels were created using Inkscape. ANOVA, analysis of variance; cAMP, cyclic adenosine monophosphate; HSP90, heat shock protein 90; IHH, immortalized human hepatocyte; ITPA, inosine triphosphate pyrophosphatase; LC‐MS/MS, liquid chromatography–tandem mass spectrometry; MOCK, mock‐transfected control; MTX, methotrexate; NF‐κB, nuclear factor‐κB; UT, untreated; p65‐NF‐κB, p65 subunit of nuclear factor‐κB; PKA‐Cα, protein kinase A catalytic subunit α. Color figure can be viewed in the online issue, which is available at http://onlinelibrary.wiley.com/doi/10.1002/art.70097/abstract.

To investigate the mechanisms underlying the differential response to MTX therapy between patients with JIA with high and low ITPA activity, IHH cells overexpressing ITPA (RE (2^−ΔΔCt^) ± SEM: 18.94 ± 5.17) and the MOCK control were treated with a range of MTX concentrations, and MTT assay was performed to compare cytotoxicity in the two cell lines. ITPA activity in IHH ITPA and IHH MOCK cell lines is shown in Supplementary Figure 5. As shown in Figure 2A, after 72 hours of treatment, ITPA‐overexpressing cells were more sensitive to MTX (25 nM, 200 nM, 600 nM) than MOCK cells (IC_50_ MOCK ± SEM: 35.6 ± 72.4 nM, IC_50_ ITPA ± SEM: 7.2 ± 5.39 nM).

### Elevated intracellular cAMP levels and PKA C‐α expression in ITPA‐overexpressing cells

MTX concentrations for subsequent experiments were selected among those showing statistical significance in cytotoxicity assay and approximating in vivo patient levels. ITPA, by hydrolyzing ITP into IMP, which is subsequently converted to AMP, could enhance AMP‐to‐adenosine conversion. Therefore, considering the potential influence of ITPA activity on adenosine levels and subsequent ADORA_2A_‐mediated signaling, we first assessed the expression of ADORA_2A_ under basal conditions in both cell lines (IHH ITPA RE^−2ΔCt^: 0.0059 ± 0.00044, IHH MOCK RE^−2ΔCt^: 0.0056 ± 0.00088, Welch's *t*‐test *P* = 0.76). Given that ADORA_2A_ activation increases intracellular cAMP and subsequently activates protein kinase A (PKA), we measured cAMP levels after MTX treatment. Although no differences in cAMP levels were observed after 24 hours of treatment, intracellular cAMP levels were higher in IHH ITPA than in the MOCK cell line at both concentrations of MTX after 48 hours (Figure 2B and C).

Based on elevated intracellular cAMP levels, the expression of the catalytic subunit Cα (PKA‐Cα) was measured to evaluate potential modulation of the PKA pathway. As shown in Figure 2D–E, no changes were observed after 24 and 48 hours. However, IHH ITPA exhibited higher PKA‐Cα expression after 72‐hour treatment with 25 nM and 200 nM MTX (Figure 2F).

### Phosphorylated p65 in IHH ITPA and MOCK cell lines

Considering the role of NF‐κB in regulating cell proliferation and inflammation, Western blot analyses were performed to assess phosphorylated p65, a subunit of NF‐κB, following MTX treatment. After 24 and 48 hours of MTX treatment, no statistically significant differences in phosphorylated p65 were observed at the tested concentrations (Figure 2G–H). Nonetheless, after 72 hours, IHH MOCK cells showed higher levels of phosphorylated p65 compared with IHH ITPA cells treated with the same concentrations of MTX (Figure 2I).

### Adenosine, inosine, hypoxanthine, and uric acid quantification in cell supernatant by LC‐MS/MS

To quantify adenosine released by the two cell lines, cell supernatants were analyzed through LC‐MS/MS. After 48 hours of treatment, analysis showed IHH MOCK cells had higher extracellular adenosine levels than IHH ITPA at 200 nM MTX (Figure 2L).

Because adenosine is rapidly converted into inosine and subsequently degraded to hypoxanthine and then uric acid, these metabolites were also measured. IHH ITPA cells exhibited significantly higher hypoxanthine levels compared with MOCK at all conditions (Figure 2N), whereas no significant differences were observed in inosine and uric acid concentrations (Figure 2M–O).

## DISCUSSION

We analyzed data from 195 patients with JIA treated with MTX to further characterize the relationship between ITPA activity and clinical remission. Remission frequency was similar to those reported in literature.^16^, ^17^

Inter‐site differences in ITPA activity are consistent with previous multicenter pharmacogenetic studies, in which changes often reflect population stratification and varying genetic backgrounds of the recruited cohorts.^18^ To account for this variability, all subsequent analyses were adjusted for study site as a covariate, following established recommendations.^19^

Consistent with literature,^11^ patients with JIA with low ITPA activity had a lower probability of reaching clinical remission than those with higher activity. ITPA activity correlates with treatment response even when measured before MTX treatment. To our knowledge, this is the first study demonstrating the clinical use of measuring ITPA activity before MTX start. Based on these findings, we identified thresholds that may help divide responders and nonresponders. Despite moderate sensitivity and specificity, both thresholds support the potential predictive value of ITPA activity. However, integration with other biomarkers or clinical factors may be necessary for clinical usefulness.

To investigate inter‐individual differences in ITPA activity, *ITPA* rs1127354 SNP, a nonsynonymous variant previously associated with reduced enzymatic activity, was genotyped.^20^, ^21^ Consistent with previous findings,^14^ ITPA activity was higher in WT patients with JIA than in those carrying the variant allele. Indeed, P32T mutation alters the enzyme's structure, resulting in a reduced activity.^22^ None of the variant allele carriers achieved clinical remission, suggesting a strong association between rs1127354 genotype and remission. Similar findings were reported in a Dutch cohort, in which WT patients showed a better response.^23^ However, 48% of WT patients also did not reach remission. Among WT patients, those with higher ITPA activity had a higher probability of remission. These results suggest that both rs1127354 genotype and ITPA activity influence remission likelihood, highlighting ITPA activity as a potentially stronger predictor than genotyping alone.

IHH cells were selected to investigate mechanisms underlying the clinical observations primarily for their nontumoral phenotype and high transfection efficiency. The use of a hepatocyte cell line is consistent with a previous study using HepG2 to investigate the interaction between methylenetetrahydrofolate reductase function and MTX treatment.^24^

The increased cytotoxicity observed in ITPA‐overexpressing cells is in line with our hypothesis that enhanced ITP to IMP conversion by ITPA may feed into adenosine synthesis pathways because IMP can be converted to AMP and subsequently to adenosine.^11^, ^25^ This could provide a direct molecular link between ITPA enzymatic function and MTX's established anti‐inflammatory mechanism through adenosine accumulation. Subsequent experiments used MTX at 25 nM and 200 nM, selected among those that showed significance in the cytotoxicity assay because they better represent levels typically reached in vivo after oral administration.^26^, ^27^ These concentrations were selected to represent physiologically relevant exposure ranges observed in vivo rather than to mimic a specific post‐dose time point.

**Figure 3:**
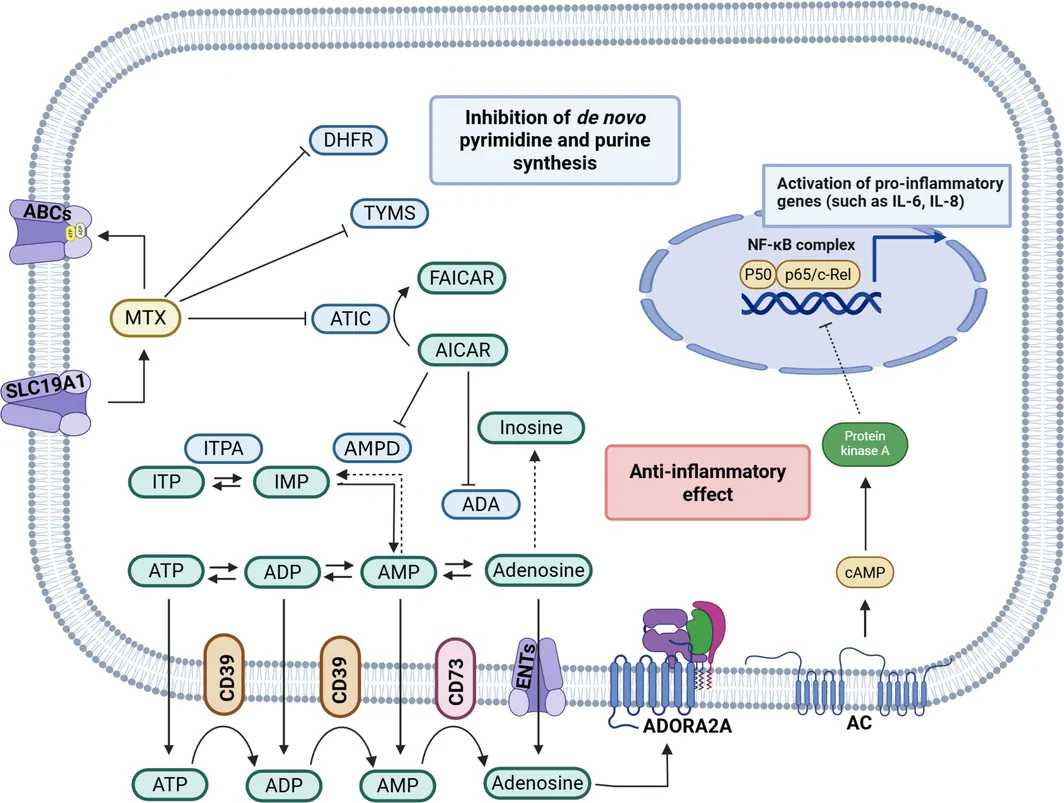
MTX is transported into the intracellular space by SLC19A1, whereas ABCs regulate its efflux. Intracellularly, MTX inhibits DHFR and TYMS, interfering with de novo pyrimidine and purine synthesis, and inhibits ATIC, promoting adenosine release. Specifically, AICAR accumulation, resulting from MTX interference with FAICAR formation, leads to the inhibition of ADA and AMPD. This blocks both adenosine degradation to inosine and AMP conversion to IMP. Moreover, ITPA‐mediated conversion of ITP to IMP could enhance AMP formation. AMP is also produced by the dephosphorylation of ATP and ADP, further increasing adenosine levels, which are transported extracellularly by ENTs. Adenosine nucleotides are also transported extracellularly, where they are converted sequentially to adenosine by CD39 and CD73. Extracellular adenosine binds to ADORA_2A_, activating adenylate cyclase, increasing cAMP, and subsequently activating PKA. PKA exerts an inhibitory effect on the nuclear NF‐κB complex, preventing the transcription of pro‐inflammatory genes (Created with BioRender.com). ADORA_2__A_, adenosine A_2_A receptor; ABC, ATP‐binding cassette transporter; ADA, adenosine deaminase; ADP, adenosine diphosphate; ADORA_2A_, adenosine A_2_A receptor; AICAR, 5‐aminoimidazole‐4‐carboxamide ribonucleotide; AMPD, AMP deaminase; ATIC, 5‐Aminoimidazole‐4‐carboxamide ribonucleotide transformylase; CD39, ectonucleoside triphosphate diphosphohydrolase‐1; CD73, ecto‐5′‐nucleotidase; DHFR, dihydrofolate reductase; ENTs, equilibrative nucleoside transporters; FAICAR, 5‐formamidoimidazole‐4‐carboxamide ribonucleotide; IMP, inosine monophosphate; ITP, inosine triphosphate; ITPA, inosine‐triphosphate‐pyrophosphatase; MTX, methotrexate; NF‐κB, nuclear factor‐κB; PKA, protein kinase A; SLC19A1, folate transporter 1; TYMS, thymidylate synthase. Color figure can be viewed in the online issue, which is available at http://onlinelibrary.wiley.com/doi/10.1002/art.70097/abstract.

After intracellular accumulation, adenosine is transported extracellularly by the equilibrative nucleoside transporters (ENTs) and binds to ADORA receptors, mainly ADORA_2A,_ mediating anti‐inflammatory effects by increasing cAMP and activating PKA, which modulates NF‐κB activity, a key regulator of inflammatory responses (Figure 3).^3^, ^4^, ^5^, ^6^, ^28^, ^29^, ^30^, ^31^ Consistent with previous studies,^32^ the observed increase in intracellular cAMP and PKA‐Cα levels in IHH ITPA cells may result from the interaction of adenosine with ADORA_2A_ receptors.

Extracellular adenosine can also be generated via sequential dephosphorylation of ATP, adenosine diphosphate, and AMP by ectoenzymes ectonucleoside triphosphate phosphohydrolase (CD39) and ecto‐5′nucleotidase (CD73) at the cell surface. In RA, both enzymes have emerged as key markers of MTX effect. Although CD73 expression is restored by MTX treatment, contributing to its anti‐inflammatory action, low CD39 density on Treg cells predicts MTX nonresponse before treatment initiation.^3^, ^33^ Although reported primarily in RA, these findings support a shared adenosine‐mediated anti‐inflammatory mechanism of MTX. In this context, we suggest ITPA activity as an additional intracellular determinant of adenosine availability, potentially acting in parallel with extracellular nucleotide catabolism.

Given the key role of NF‐κB in modulating the inflammatory responses,^34^ we evaluated total and phosphorylated p65 protein expression. The higher activation of p65 may underlie the reduced MTX cytotoxicity in the IHH MOCK cell line, consistent with the role of NF‐κB in apoptosis and cell survival.^34^, ^35^ Moreover, these findings align both with previous observations in patients with JIA^11^ and in the present cohort, in which higher ITPA activity has been associated with an increased likelihood of achieving clinical remission.

Because phosphorylated p65 is not sufficient to confirm NF‐κB activation, investigating the nuclear translocation of the subunit will be essential to obtain a more accurate measurement of the complex activity. Nevertheless, although NF‐κB nuclear translocation primarily depends on degradation of the inhibitor of κB, the observed increase in p65 Ser536 phosphorylation, often associated with enhanced transcriptional activity,^36^, ^37^ strongly indicates the complex activation.

Although analysis showed increased adenosine levels in IHH MOCK cells supernatant after the highest MTX concentration, hypoxanthine resulted in elevated IHH ITPA under all conditions, including untreated cells. Following MTX‐induced intracellular accumulation, adenosine is transported extracellularly via ENTs and rapidly metabolized to inosine by membrane‐associated adenosine deaminase (ecto–adenosine deaminase), then to hypoxanthine by purine nucleoside phosphorylase, and further metabolized to uric acid by xanthine oxidase.^3^, ^38^, ^39^, ^40^, ^41^, ^42^ Because the stop solution was added 30 minutes before the end of the incubation, the already released adenosine could have been metabolized to inosine and subsequently to hypoxanthine, suggesting a faster and greater release of adenosine by IHH ITPA cells, followed by its degradation.

However, increased hypoxanthine might also result from the conversion of inosine metabolized from IMP, the product of ITP conversion by ITPA, which can be converted to AMP or degraded to inosine.^25^, ^43^ Because inosine is an anti‐inflammatory in different disease models, it might underlie the differences between the two cell lines. Indeed, a study on a human epithelial cell line demonstrated that inosine suppressed interleukin‐8 production, and several studies demonstrated its ability to decrease tumor necrosis factor‐α in vitro and in vivo.^44^, ^45^, ^46^ Inosine acts both independently and via ADORA receptors, especially ADORA_2A_.^45^, ^47^ Its affinity for ADORA_2A_ may explain the results obtained in IHH ITPA cells because the binding of inosine may activate anti‐inflammatory signaling pathways similar to those induced by adenosine.

In a murine pleurisy model, adenosine and inosine at sub‐effective doses showed a synergistic anti‐inflammatory effect, reducing leukocyte migration and neutrophil infiltration, probably through ADORA_2A_ and ADORA_2B_ receptors.^48^ Thus, inosine and adenosine might act synergistically in IHH ITPA cells, potentially reflecting what happens in patients with JIA with high ITPA activity.

Beyond the adenosine/inosine‐related pathway, other explanations warrant consideration. An alternative hypothesis is that ITPA activity influences MTX response through ITP‐MTX competition for folylpolyglutamate synthetase.^49^, ^50^ Specifically, low ITPA activity leads to ITP accumulation, potentially interfering with MTX polyglutamate formation, reducing intracellular active MTX metabolites and diminishing therapeutic efficacy. Conversely, high ITPA activity would efficiently clear ITP, removing this competitive inhibition, allowing optimal MTX polyglutamation, enhancing target enzymes inhibition, and improving therapeutic outcomes. This alternative mechanism provides a plausible biochemical explanation for our clinical observations.

Although the findings are promising, there are several limitations that should be considered. These include a relatively small sample size and single ancestry cohort considered to calculate ITPA activity cutoffs, with no replication cohort currently available. Additionally, the lack of racial diversity among patients may limit the generalizability of the findings. Increasing patient numbers in future studies could help find a more reliable cutoff. Despite sensitivity and specificity limitations, this is the first study to define cutoffs for ITPA activity in JIA, representing a crucial step toward translating this biomarker into clinical practice. Moreover, our study did not investigate the correlation between MTX polyglutamate levels and ITPA activity, which represents a limitation in establishing the mechanistic basis for the clinical findings.

In vitro limitations include the limited number of biologic replicates and the use of a hepatocyte cell line, which does not fully represent the cell types involved in the pathophysiology of the disease. The aim was to explore the molecular mechanisms associated with ITPA activity independently of the disease context, and this model provided a controlled system to isolate the effects of the different ITPA activity on MTX response. Repeating these experiments in a disease‐representative cell model will help confirm the observed differences and clarify their impact in JIA.

Single‐time point adenosine measurement limits the understanding of its dynamic metabolism. Future studies considering multiple time points would provide a more comprehensive understanding of adenosine and inosine anti‐inflammatory effects, as well as treatment with these metabolites to clarify their individual roles in modulating the anti‐inflammatory effect. The use of selective antagonists could validate ADORA_2A_ involvement.

In conclusion, the present study proposes ITPA activity as a new therapeutic tool for personalizing JIA treatment. Our findings suggest that patients with low ITPA activity, at baseline or during treatment, may be assigned to a more aggressive therapy, such as the early initiation of biologic drug. This approach could optimize treatment response and reduce the risk of disease progression and permanent joint damage.

## AUTHOR CONTRIBUTIONS

All authors contributed to at least one of the following manuscript preparation roles: conceptualization AND/OR methodology, software, investigation, formal analysis, data curation, visualization, and validation AND drafting or reviewing/editing the final draft. As corresponding author, Dr Stocco confirms that all authors have provided the final approval of the version to be published and takes responsibility for the affirmations regarding article submission (eg, not under consideration by another journal), the integrity of the data presented, and the statements regarding compliance with institutional review board/Declaration of Helsinki requirements.

## Supporting information


**Disclosure Form**:

**Data S1** Supporting Information

## References

[1] McCurdy D , Parsa MF . Updates in juvenile idiopathic arthritis. Adv Pediatr 2021;68:143–170. doi:10.1016/j.yapd.2021.05.014 34243850

[2] Ravelli A , Martini A . Juvenile idiopathic arthritis. Lancet 2007;369(9563):767–778. doi:10.1016/S0140-6736(07)60363-8 17336654

[3] Cronstein BN , Aune TM . Methotrexate and its mechanisms of action in inflammatory arthritis. Nat Rev Rheumatol 2020;16(3):145–154. doi:10.1038/s41584-020-0373-9 32066940

[4] Brown PM , Pratt AG , Isaacs JD . Mechanism of action of methotrexate in rheumatoid arthritis, and the search for biomarkers. Nat Rev Rheumatol 2016;12(12):731–742. doi:10.1038/nrrheum.2016.175 27784891

[5] Vincenzi F , Padovan M , Targa M , et al. A(2A) adenosine receptors are differentially modulated by pharmacological treatments in rheumatoid arthritis patients and their stimulation ameliorates adjuvant‐induced arthritis in rats. PLoS One 2013;8(1):e54195. doi:10.1371/journal.pone.0054195 23326596 PMC3543361

[6] Minguet S , Huber M , Rosenkranz L , et al. Adenosine and cAMP are potent inhibitors of the NF‐kappa B pathway downstream of immunoreceptors. Eur J Immunol 2005;35(1):31–41. doi:10.1002/eji.200425524 15580656

[7] Csóka B , Haskó G . Adenosine, inflammation pathways and therapeutic challenges. Joint Bone Spine 2011;78(1):4–6. doi:10.1016/j.jbspin.2010.08.010 20889361

[8] Montesinos MC , Yap JS , Desai A , et al. Reversal of the antiinflammatory effects of methotrexate by the nonselective adenosine receptor antagonists theophylline and caffeine: evidence that the antiinflammatory effects of methotrexate are mediated via multiple adenosine receptors in rat adjuvant arthritis. Arthritis Rheum 2000;43(3):656–663. doi:10.1002/1529-0131(200003)43:3<656:AID-ANR23>3.0.CO;2-H 10728760

[9] Nesher G , Mates M , Zevin S . Effect of caffeine consumption on efficacy of methotrexate in rheumatoid arthritis. Arthritis Rheum 2003;48(2):571–572. doi:10.1002/art.10766 12571869

[10] Bava C , Mongelli F , Pistorio A , et al. A prediction rule for lack of achievement of inactive disease with methotrexate as the sole disease‐modifying antirheumatic therapy in juvenile idiopathic arthritis. Pediatr Rheumatol Online J 2019;17(1):50. doi:10.1186/s12969-019-0355-0 31345226 PMC6657374

[11] Pastore S , Stocco G , Moressa V , et al. 5‐Aminoimidazole‐4‐carboxamide ribonucleotide‐transformylase and inosine‐triphosphate‐pyrophosphatase genes variants predict remission rate during methotrexate therapy in patients with juvenile idiopathic arthritis. Rheumatol Int 2015;35(4):619–627. doi:10.1007/s00296-014-3131-y 25240429

[12] Zamzami MA . Inosine triphosphate pyrophosphatase (ITPase): functions, mutations, polymorphisms and its impact on cancer therapies. Cells 2022;11(3):384. doi:10.3390/cells11030384 35159194 PMC8833965

[13] Wallace CA , Ruperto N , Giannini E ; Childhood Arthritis and Rheumatology Research Alliance ; Pediatric Rheumatology International Trials Organization ; Pediatric Rheumatology Collaborative Study Group. Preliminary criteria for clinical remission for select categories of juvenile idiopathic arthritis. J Rheumatol 2004;31(11):2290–2294. 15517647

[14] Shipkova M , Lorenz K , Oellerich M , et al. Measurement of erythrocyte inosine triphosphate pyrophosphohydrolase (ITPA) activity by HPLC and correlation of ITPA genotype‐phenotype in a Caucasian population. Clin Chem 2006;52(2):240–247. doi:10.1373/clinchem.2005.059501 16384889

[15] Pelin M , De Iudicibus S , Fusco L , et al. Role of oxidative stress mediated by glutathione‐s‐transferase in thiopurines’ toxic effects. Chem Res Toxicol 2015;28(6):1186–1195. doi:10.1021/acs.chemrestox.5b00019 25928802

[16] Bardan I , Fagerli KM , Sexton J , et al. Treatment response to tumor necrosis factor inhibitors and methotrexate monotherapy in adults with juvenile idiopathic arthritis: data from NOR‐DMARD. J Rheumatol 2023;50(4):538–547. doi:10.3899/jrheum.220645 36379571

[17] Castillo‐Vilella M , Giménez N , Tandaipan JL , et al. Clinical remission and subsequent relapse in patients with juvenile idiopathic arthritis: predictive factors according to therapeutic approach. Pediatr Rheumatol Online J 2021;19(1):130. doi:10.1186/s12969-021-00607-0 34419078 PMC8380331

[18] Zhou Y , Lauschke VM . Population pharmacogenomics: an update on ethnogeographic differences and opportunities for precision public health. Hum Genet 2022;141(6):1113–1136. doi:10.1007/s00439-021-02385-x 34652573 PMC9177500

[19] Kahan BC , Rushton H , Morris TP , et al. A comparison of methods to adjust for continuous covariates in the analysis of randomised trials. BMC Med Res Methodol 2016;16(1):42. doi:10.1186/s12874-016-0141-3 27068456 PMC4827223

[20] Sumi S , Marinaki AM , Arenas M , et al. Genetic basis of inosine triphosphate pyrophosphohydrolase deficiency. Hum Genet 2002;111(4‐5):360–367. doi:10.1007/s00439-002-0798-z 12384777

[21] Arenas M , Duley J , Sumi S , et al. The ITPA c.94C>A and g.IVS2+21A>C sequence variants contribute to missplicing of the ITPA gene. Biochim Biophys Acta 2007;1772(1):96–102. doi:10.1016/j.bbadis.2006.10.006 17113761

[22] Stenmark P , Kursula P , Flodin S , et al. Crystal structure of human inosine triphosphatase. Substrate binding and implication of the inosine triphosphatase deficiency mutation P32T. J Biol Chem 2007;282(5):3182–3187. doi:10.1074/jbc.M609838200 17138556

[23] Wessels JA , Kooloos WM , De Jonge R , et al. Relationship between genetic variants in the adenosine pathway and outcome of methotrexate treatment in patients with recent‐onset rheumatoid arthritis. Arthritis Rheum 2006;54(9):2830–2839. doi:10.1002/art.22032 16947783

[24] Wang YC , Wu MT , Tang FY , et al. *MTHFR* C677T polymorphism increases MTX sensitivity via the inhibition of *S*‐adenosylmethionine and *de novo* purine synthesis. Clin Sci (Lond) 2019;133(2):253–267. doi:10.1042/CS20180932 30606816

[25] Pareek V , Pedley AM , Benkovic SJ . Human de novo purine biosynthesis. Crit Rev Biochem Mol Biol 2021;56(1):1–16. doi:10.1080/10409238.2020.1832438 33179964 PMC7869020

[26] Carmichael SJ , Beal J , Day RO , et al. Combination therapy with methotrexate and hydroxychloroquine for rheumatoid arthritis increases exposure to methotrexate. J Rheumatol 2002;29(10):2077–2083. 12375315

[27] Xu K , Cai Y‐S , Lu S‐M , et al. Autophagy induction contributes to the resistance to methotrexate treatment in rheumatoid arthritis fibroblast‐like synovial cells through high mobility group box chromosomal protein 1. Arthritis Res Ther 2015;17(1):374. doi:10.1186/s13075-015-0892-y 26702616 PMC4718027

[28] Baldwin SA , Beal PR , Yao SYM , et al. The equilibrative nucleoside transporter family, SLC29. Pflugers Arch 2004;447(5):735–743. doi:10.1007/s00424-003-1103-2 12838422

[29] da Silva JLG , Passos DF , Bernardes VM , et al. ATP and adenosine: role in the immunopathogenesis of rheumatoid arthritis. Immunol Lett 2019;214:55–64. doi:10.1016/j.imlet.2019.08.009 31479688

[30] Fredholm BB , Chern Y , Franco R , et al. Aspects of the general biology of adenosine A2A signaling. Prog Neurobiol 2007;83(5):263–276. doi:10.1016/j.pneurobio.2007.07.005 17804147

[31] Dell'Acqua ML , Scott JD . Protein kinase A anchoring. J Biol Chem 1997;272(20):12881–12884. doi:10.1074/jbc.272.20.12881 9190362

[32] Mediero A , Perez‐Aso M , Cronstein BN . Activation of adenosine A(2A) receptor reduces osteoclast formation via PKA‐ and ERK1/2‐mediated suppression of NFκB nuclear translocation. Br J Pharmacol 2013;169(6):1372–1388. doi:10.1111/bph.12227 23647065 PMC3831714

[33] Bossennec M , Rodriguez C , Hubert M , et al. Methotrexate restores CD73 expression on Th1.17 in rheumatoid arthritis and psoriatic arthritis patients and may contribute to its anti‐inflammatory effect through ado production. J Clin Med 2019, 3;8(11):1859. doi:10.3390/jcm8111859 31684171 PMC6912794

[34] Liu T , Zhang L , Joo D , et al. NF‐κB signaling in inflammation. Signal Transduct Target Ther 2017;2(1):17023. doi:10.1038/sigtrans.2017.23 29158945 PMC5661633

[35] Verzella D , Pescatore A , Capece D , et al. Life, death, and autophagy in cancer: NF‐κB turns up everywhere. Cell Death Dis 2020;11(3):210. doi:10.1038/s41419-020-2399-y 32231206 PMC7105474

[36] Sasaki CY , Barberi TJ , Ghosh P , et al. Phosphorylation of RelA/p65 on serine 536 defines an IkappaBalpha‐independent NF‐kappaB pathway. J Biol Chem 2005;280(41):34538–34547. doi:10.1074/jbc.M504943200 16105840

[37] Geng B , Chen X , Chi J , et al. Platelet membrane‐coated alterbrassicene A nanoparticle inhibits calcification of the aortic valve by suppressing phosphorylation P65 NF‐κB. Theranostics 2023;13(11):3781–3793. doi:10.7150/thno.85323 37441596 PMC10334836

[38] Löfgren L , Pehrsson S , Hägglund G , et al. Accurate measurement of endogenous adenosine in human blood. PLoS One 2018;13(10):e0205707. doi:10.1371/journal.pone.0205707 30359421 PMC6201894

[39] Allard B , Allard D , Buisseret L , et al. The adenosine pathway in immuno‐oncology. Nat Rev Clin Oncol 2020;17(10):611–629. doi:10.1038/s41571-020-0382-2 32514148

[40] Zoref‐Shani E , Kessler‐Icekson G , Sperling O . Pathways of adenine nucleotide catabolism in primary rat cardiomyocyte cultures. J Mol Cell Cardiol 1988;20(1):23–33. doi:10.1016/S0022-2828(88)80176-7 3259263

[41] Garcia‐Gil M , Camici M , Allegrini S , et al. Metabolic aspects of adenosine functions in the brain. Front Pharmacol 2021;12:672182. doi:10.3389/fphar.2021.672182 34054547 PMC8160517

[42] Haskó G , Sitkovsky MV , Szabó C . Immunomodulatory and neuroprotective effects of inosine. Trends Pharmacol Sci 2004;25(3):152–157. doi:10.1016/j.tips.2004.01.006 15019271

[43] Chen W , Hoerter J , Guéron M . A comparison of AMP degradation in the perfused rat heart during 2‐deoxy‐D‐glucose perfusion and anoxia. Part I: the release of adenosine and inosine. J Mol Cell Cardiol 1996;28(10):2163–2174. doi:10.1006/jmcc.1996.0208 8930811

[44] Marton A , Pacher P , Murthy KG , et al. Anti‐inflammatory effects of inosine in human monocytes, neutrophils and epithelial cells in vitro. Int J Mol Med 2001;8(6):617–621. doi:10.3892/ijmm.8.6.617 11712075

[45] Lovászi M , Németh ZH , Gause WC , et al. Inosine monophosphate and inosine differentially regulate endotoxemia and bacterial sepsis. FASEB J 2021;35(11):e21935. doi:10.1096/fj.202100862R 34591327 PMC9812230

[46] Gomez G , Sitkovsky MV . Differential requirement for A2a and A3 adenosine receptors for the protective effect of inosine in vivo. Blood 2003;102(13):4472–4478. doi:10.1182/blood-2002-11-3624 12947007

[47] Welihinda AA , Kaur M , Raveendran KS , et al. Enhancement of inosine‐mediated A_2A_R signaling through positive allosteric modulation. Cell Signal 2018;42:227–235. doi:10.1016/j.cellsig.2017.11.002 29126977 PMC5732040

[48] da Rocha Lapa F , da Silva MD , de Almeida Cabrini D , et al. Anti‐inflammatory effects of purine nucleosides, adenosine and inosine, in a mouse model of pleurisy: evidence for the role of adenosine A2 receptors. Purinergic Signal 2012;8(4):693–704. doi:10.1007/s11302-012-9299-2 22456813 PMC3486164

[49] Baram J , Chabner BA , Drake JC , et al. Identification and biochemical properties of 10‐formyldihydrofolate, a novel folate found in methotrexate‐treated cells. J Biol Chem 1988;263(15):7105–7111. doi:10.1016/S0021-9258(18)68611-9 3366769

[50] Allegra CJ , Chabner BA , Drake JC , et al. Enhanced inhibition of thymidylate synthase by methotrexate polyglutamates. J Biol Chem 1985;260(17):9720–9726. doi:10.1016/S0021-9258(17)39298-0 2410416

