# Supplementary material for: Inosine‐Triphosphate‐Pyrophosphatase Activity as a Potential Predictor of Methotrexate Remission in Juvenile Idiopathic Arthritis
Source: Arthritis Rheumatol. 2026 Mar 22;78(9):1989–99. doi: 10.1002/art.70097 (PMC13507606; doi:10.1002/art.70097)
Supplement: Supplementary file 2 [file ART-78-1989-s002.docx]

Supplementary Material

*Measurement of ITPA activity*

Sample preparation consists in adding 150 µL dithiothreitol 0.01M (Sigma-Aldrich, Milan, Italy), 10 µL Tris-HCl 0.1M (pH=9) (T1503, Sigma-Aldrich, Italy) and 10 µL MgCl_2_ 1M (M8266, Sigma-Aldrich, Italy) to patient’s erythrocytes lysate (1:6 in MilliQ water). Then, after a 5 min incubation at 37°C, 10 µL ITP 40 mM (Sigma-Aldrich, Milan, Italy) was added, and the samples were incubated for 15 min at 37°C. The enzymatic reaction was stopped by adding 20 µL perchloric acid 4M (Sigma-Aldrich, Milan, Italy), followed by incubation at -20°C for 10 min. Subsequently, 30 µL dipotassium dihydrogen phosphate (Sigma-Aldrich, Milan, Italy) was added, and the samples were centrifuged at 13,000 *g* for 10 minutes. Twenty µL of supernatant were injected in the instrument Agilent 1200 HPLC coupled with UV detector (Agilent Technologies, Milan) for the detection of IMP within 24 hours after preparation. For the analysis of ITPA enzyme activity, a 0.02 M acid phosphate buffer with pH 2.5 (mobile phase A) and acetonitrile (mobile phase B) were used as mobile phases. A reversed-phase analytical column (Aqua Perfect C18 with 5 μm pores, length of 250 mm and diameter of 4.0 mm) was used for analysis with a pre-column (Zorbax SB-Aq with 5 μm pores, length of 12.5 mm and diameter of 4.6 mm) to retain any impurities in the sample. The flow rate was set at 1.1 ml/minute and kept constant during the run conducted at room temperature. In detail, the gradient began with 100% mobile phase A. Then, mobile phase A was linearly decreased to 97% over 8 minutes. Subsequently, A was decreased to 20% over the next 1 minute (from 8 to 9 minutes) and then held at 20% until 9.10 minutes. The UV wavelength was set at 262 nm for the entire run duration, which was 11 minutes. The IMP calibration line consisted of 6 standard solutions distributed in a concentration range of 5.8 μM to 1500 μM prepared with appropriate dilutions in Milli-Q water from the IMP stock solution (Sigma Aldrich, Milan, Italy), after evaluating the absence of matrix signal interference in the retention times of the analytes. The last minutes, the column was reconditioned. ITPA activity is expressed as nmol of IMP per hour (nmol IMP/h).

*Immortalized cell lines and cell culture*

IHH cell lines were cultured in Dulbecco's modified Eagle's medium High Glucose (DMEM-HG) (EuroClone, Milan, Italy) supplemented with 10% fetal bovine serum (FBS) (Sigma-Aldrich, Milan, Italy), 1.5% of Hepes Buffer 1M (Sigma-Aldrich, Milan, Italy), 1.25% L-glutamine 200 mM (EuroClone, Milano Italy), 1% penicillin 10000 UI/mL (Euroclone, Milan, Italy) and 1% of streptomycin 10 mg/mL (Euroclone, Milan, Italy), 0.01% human insulin 10^-4^ M (Sigma-Aldrich, Milan, Italy) and 0.04% dexamethasone 1 mg/mL (DEX) (Sigma-Aldrich, Milan, Italy), with the latter added only at the time of use. The cell lines were incubated in a humidified atmosphere at 37 °C with 5% CO_2_ and cell passage was performed when the cells reached approximately 80% confluence.

*MTT cytotoxicity test*

Four hours before the end of the incubation, 20 µL of MTT (5 mg/ml in phosphate buffer saline) (Sigma-Aldrich, Milan, Italia) were added to each well. At the end of the incubation period, the formazan product was dissolved in 100 µL di dimethyl sulfoxide (DMSO) (Sigma-Aldrich, Milano, Italia). Absorbance was measured at 540 nm, with background subtraction at 630 nm, using the Automated Microplate Reader EL311 (BIO-TEK® Instruments, Vermont, USA). Cell viability was expressed as percentage relative to untreated control cells.

*Quantitative real‑time PCR*

Thermal cycling conditions for Sybr® green were as follow: 30 s at 98°C, followed by 40 cycles at 95°C for 5 s and 60°C for 1 min. A melting curve analysis was performed at the end to confirm the specificity of the amplification. Relative expression levels were evaluated using the comparative Ct method (2^-ΔCt^ and 2^-ΔΔCt^)^1^. ADORA_2A_ expression values were normalized to the housekeeping gene GAPDH.

*Western blot analysis*

After the incubation period, cell pellets were collected, washed in cold PBS, and lysed using RIPA buffer (Thermo Fisher, Milan, Italy) containing the Halt™ Protease Inhibitor Cocktail (100X) (ThermoFisher Scientific, Milan, Italy), sodium fluoride 5 mM (Sigma-Aldrich, Milan, Italy), and sodium orthovanadate 1 mM (Sigma-Aldrich, Milan, Italy). Protein quantification was performed using the Pierce™ BCA Protein Assay Kit (TermoFisher Scientific, Milan, Italy), following the manufacturer’s instructions.

Ten µg of proteins were separated on Sodium Dodecyl Sulphate – Polyacrylamide Gel Electrophoresis (SDS-PAGE) Bolt 4-12% Bis-Tris Plus Gels (Thermo Fisher Scientific, Milan, Italy) and transblotted onto nitrocellulose membranes (Thermo Fisher Scientific, Milan, Italy). SeeBlue™ Plus2 Pre-stained Protein Standard (Thermo Fisher Scientific, Milan, Italy) was included in each gel to allow protein identification and membrane cutting. After transfer, protein loading was verified using Ponceau S staining solution (Thermo Fisher Scientific, Milan, Italy). Membranes were cut according to molecular weight to allow simultaneous incubation with multiple primary antibodies, and subsequently were blocked with 5% milk in Tris-buffered saline buffer (TTBS) (0.012 % p/V Trizma (Merck, Saint Louis, USA); 0.009 % p/V NaCl (Merck, Saint Louis, USA) completed with 0.001% Tween 20 (Merck, Saint Louis, MO, USA) for 1 hour. Subsequently, membranes were incubated overnight at 4°C with primary antibodies: rabbit anti-HSP90 (1:1000, Cell Signaling, Danvers, USA), mouse anti-GAPDH (1:1000, Affinity, USA), rabbit anti-ITPA (1:800, Abcam, Cambridge, UK), rabbit anti-PKA C-α (1:1000, Cell Signaling, Daners, USA), mouse anti-p65-NFκB (1:500, Santa Cruz Biotechnology, Dallas, USA), rabbit anti-p-p65-NFκB (1:1000. Cell Signaling, Danvers, USA).

After overnight incubation, membranes were washed and incubated with the appropriate secondary antibody: anti-mouse (1:8000 dilution, Cell Signaling, Danvers, USA), anti-rabbit (1:10000 dilution, Merck, Saint Louis, USA). Chemiluminescence was detected using the LiteABLOT Turbo Chemiluminescent Substrate (EuroClone, Milan, Italy) and visualized with the ChemiDoc™ MP Imaging System (Bio-Rad Laboratories). All membranes were analysed using ImageJ® software.

To quantify total p65 and PKA, membranes were stripped using ReBlot Plus Mild Antibody Stripping Solution, 10x (Millipore, Milan, Italy), according to manufacturer’s instructions, and subsequently incubated with the appropriate primary antibody.

*Quantification of adenosine, inosine, hypoxanthine and uric acid in cell supernatants through LC-MS/MS*

The stop solution was composed of 10 μM α,β-methyleneadenosine 5′-diphosphate (AOPCP) (Sigma-Aldrich, Milan, Italy), an inhibitor of ecto-5’-nucleosidasi (CD73) inhibitor, 10 μM erythro-9-(2-hydroxy-3-nonyl)adenine (EHNA) (Sigma-Aldrich, Milan, Italy), an adenosine deaminase (ADA) inhibitor, and 10 μM dipyridamole (DIP) (Sigma-Aldrich, Milan, Italy), which blocks adenosine reuptake^2, 3^.

The compounds separation was performed on an Acclaim PepMap RSLC C18 column (150 mm x 1 mm, particle size 2 µm Thermo Fisher Scientific) using an aqueous phase (A) of 0.1% formic acid and an organic phase (B) of acetonitrile 0.1% formic acid in acetonitrile. The flow rate was set at 80 µL/min; the linear gradient was increased from 1% B to 60% B in 9 min, from 60% B to 100% B in 2 minutes, and it was followed by column washing and equilibration. The total running time was 13.5 min. The column oven was set at 35°C, the autosampler was set at 7 °C and the injection volume was 2 µL. The data acquisition was carried out with the software Xcalibur (Thermo Fisher Scientific, Milan, Italy). The ESI source operated in positive mode and the following parameters were set: ion transfer tube temperature 300 °C, spray voltage 3200 V, sheath gas and auxiliary gas were set to 35 and 10 (arbitrary units), respectively. Acquisition parameters for master scan were as follows: scan range 100–1000 m/z, 90,000 resolution at m/z 200, normalized AGC target 100% and maximum injection time was set in mode auto. Parameters for data dependent MS2 were as follows: intensity threshold 2x10^4^, 5 dependent scans, isolation window (m/z) 2, stepped HCD collision energy (%) was 20, 40, 60, orbitrap resolution at m/z 200 was 60,000, normalized AGC target 100% and maximum injection time was set in mode auto. Internal calibration was performed prior to each analysis sequence. Five replicates of each biological sample were used for statistical analysis. Three technical replicates were used for quantification purposes, while data acquired in data dependent mode were used for identification only. LC-MS/MS data were processed using Compound Discoverer (v3.3, Thermo Fisher), with analyte response normalized to cell count and annotated per guideline^4^.

**Supplementary Figure 1** Boxplots showing ITPA activity measured at all timepoints divided by clinical site.

**Supplementary Figure 2** Boxplots showing ITPA activity measured at therapy onset divided by clinical site.

**Supplementary Table 1** Summary table of ITPA activity measured during therapy by clinical site.

**Supplementary Table 2** Summary Table of ITPA activity measured at therapy onset by clinical site.

**Supplementary Figure 3** Boxplots showing ITPA activity stratified by time of measurement: at therapy onset vs subsequent treatment timepoints (Subsequent).

**Supplementary Figure 4** Boxplots showing ITPA activity in patients who achieved remission stratified by time of measurement: at therapy onset vs subsequent treatment timepoints (Subsequent).

**Supplementary Figure 5** ITPA activity in IHH ITPA (402.5 ± 20.5 nmol IMP/h/1 x 10^6^ cells) and IHH MOCK (215 ± 5 nmol IMP/h/1 x 10^6^ cells) cell lines. Data are presented as mean ± SEM from two biological replicates.

References

1. Livak KJ, Schmittgen TD. Analysis of relative gene expression data using real-time quantitative PCR and the 2(-Delta Delta C(T)) method. Methods 2001;25:402-8.
2. Löfgren L, Pehrsson S, Hägglund G, et al. Accurate measurement of endogenous adenosine in human blood. PLoS One 2018;13:e0205707.
3. Shryock JC, Boykin MT, Hill JA, et al. A new method of sampling blood for measurement of plasma adenosine. Am J Physiol Heart Circ Physiol 1990;258:H1232-9.
4. Alseekh S, Aharoni A, Brotman Y, et al. Mass spectrometry-based metabolomics: a guide for annotation, quantification and best reporting practices. Nat Methods 18, 747-756.
